# Supplementary material for: Optimized Metabotype Definition Based on a Limited Number of Standard Clinical Parameters in the Population-Based KORA Study
Source: Life (Basel). 2022 Sep 20;12(10):1460. doi: 10.3390/life12101460 (PMC9604647; doi:10.3390/life12101460)
Supplement: Supplementary file 1 [file life-12-01460-s001.zip › life-1884818-supplementary.pdf]

Supporting information

Table S1: Characteristics of the KORA FF4 study population.

	Total N=2120	Men N=1034	Women N=1086	P-value
**Demographic characteristics**				
Age (years) Median (IQR)	54 (44, 64)	**55 (44, 65)**	53 (44, 63)	0.069
Education				
<10 years	139 (6.6%)	30 (2.9%)	**109 (10.0%)**	<0.001
10 to <12 years	1036 (48.9%)	446 (43.1%)	**590 (54.3%)**	
>= 12 years	940 (44.3%)	**555 (53.7%)**	385 (35.5%)	
Missing	5 (0.2%)	3 (0.3%)	2 (0.2%)	
BMI (kg/m^2) Median (IQR)	27 (24.1, 29.9)	**27 (25, 30)**	26 (23, 30)	<0.001
Normal (18.5 - < 25)	710 (33.5%)	251 (24.3%)	**459 (42.3%)**	<0.001
Overweight (25 - < 30)	896 (42.3%)	**530 (51.3%)**	366 (33.7%)	
Obese (>= 30)	505 (23.8%)	**250 (24.2%)**	255 (23.5%)	
Missing	9 (0.4%)	3 (0.3%)	6 (0.6%)	
Physical Activity				
Active	1232 (58.1%)	591 (57.2%)	**641 (59.0%)**	0.408
Inactive	888 (41.9%)	**443 (42.8%)**	445 (41.0%)	
Smoking				
Smoker	384 (16.4%)	**189 (18.3%)**	159 (14.6%)	<0.001
Ex-Smoker	880 (41.5%)	**505 (48.8%)**	375 (34.5%)	
NeverSmoker	891 (42.0%)	340 (32.9%)	**551 (50.7%)**	
**Prevalence of disease** n (%)				
Diabetes Mellitus	94 (4.4%)	**54 (5.2%)**	40 (3.7%)	0.081
Missing	132 (6.2%)	77 (7.4%)	55 (5.1%)	
Hypertension	45 (2.1%)	**35 (3.4%)**	10 (0.9%)	0.734
Missing	3 (0.1%)	2 (0.2%)	1 (0.1%)	
Hyperuricemia	45 (2.1%)	**35 (3.4%)**	10 (0.9%)	<0.001
Missing	3 (0.1%)	2 (0.2%)	1 (0.1%)	
Dyslipidemia	157 (7.4%)	**85 (8.2%)**	72 (6.6%)	0.186
Missing	3 (0.1%)	2 (0.2%)	1 (0.1%)	
Any metabolic disease	442 (20.8%)	**242 (45.7%)**	200 (18.4%)	0.002
Missing	102 (4.8%)	60 (5.8%)	42 (3.9%)	
Stroke	35 (1.7%)	16 (1.5%)	**19 (1.7%)**	0.885
Missing	29 (1.4%)	23 (2.2%)	6 (0.6%)	
Myocardial Infarction	27 (1.3%)	**21 (2.0%)**	6 (0.6%)	0.003
Missing	44 (2.1%)	35 (3.4%)	9 (0.8)	
Any cardiovascular disease	60 (2.8 %)	**35 (3.4%)**	25 (2.3%)	0.13
Missing	65 (3.1%)	50 (4.8%)	15 (1.4%)	

Median (25th, 75th percentile) for continuous variables and n (column %) for categorical variables; P values are from Kruskal–Wallis test for continuous variables and Pearson's chi-squared test for categorical variables. Prevalence: Due to missing information, reduced datasets for type 2 diabetes n=1988, hypertension n=2116, hyperuricemia n=2117, dyslipidemia n=2117, "any metabolic disease" n=2018, stroke n=2091, myocardial infraction n=2076, "any cardiovascular disease" n=2055.The highest values are marked in bold. KORA, Cooperative Health Research in the Region of Augsburg.

Table S2: Incidence of diseases in the unfavorable metabotype clusters identified in different clustering models based on combinations of seven parameters, versus the 14-parameter clustering model, KORA FF4 study.

Model	Parameters							Unfavorable Cluster	Incidence of disease									
									Diabetes	Hypertension	Hyperuricemia	Dyslipidemia	Any metabolic disease	Rank (metabolic disease)	Stroke	Myocardial Infraction	Any cardiovascular disease	Rank (cardiovascular disease)
								N						%				
	14-parameter model: TG, Uric acid, HDLc, Insulin, BMI, non-HDLc, Glucose, Alkaline-phosphatase, GGT, GOT, GPT, Glycated Hemoglobin, Leukocyte, CRP							202	26.8	11.9	5.0	12.0	51.4	14	3.6	2.1	6.0	16
1	TG	BMI	Uric Acid	Glucose	HDLc	Non-HDLc	Insulin	242	23.9	12.0	7.9	16.6	53.6	9	4.3	3.1	7.3	9
2	TG	BMI	Uric Acid	Glucose	HDLc	Non-HDLc		215	18.6	11.2	7.0	19.2	51.2	13	3.8	2.3	7.0	12
3	TG	BMI	Uric Acid	Glucose	HDLc			211	20.7	10.9	7.1	18.1	52.3	11	3.9	2.5	6.7	15
4	TG	BMI	Uric Acid	Glucose				187	22.6	10.8	6.4	19.4	56.0	7	4.3	3.0	7.5	7
5	TG	BMI	Uric Acid					197	11.3	13.3	5.6	16.3	41.7	15	4.1	2.7	7.0	11
6	TG		Uric Acid	Glucose				153	25.9	11.2	4.6	19.7	58.3	4	5.3	5.4	8.4	4
7		BMI	Uric Acid	Glucose	HDLc	Non-HDLc		222	30.2	13.6	**9.5**	17.2	**62.0**	1	4.6	2.4	7.4	8
8			Uric Acid	Glucose	HDLc	Non-HDLc		629	11.3	11.9	6.1	16.4	39.3	16	2.7	3.4	5.9	17
9	TG		Uric Acid	Glucose	HDLc	Non-HDLc		201	18.4	11.0	6.0	21.0	51.9	12	4.1	2.6	6.9	14
10	TG	BMI		Glucose	HDLc	Non-HDLc		196	27.4	11.3	5.1	19.0	56.1	6	5.2	3.2	8.8	2
11	TG	BMI	Uric Acid		HDLc	Non-HDLc		332	10.3	12.9	4.2	16.9	37.8	17	2.8	**4.3**	6.9	13
12	TG	BMI	Uric Acid			Non-HDLc		356	8.5	13.2	3.9	17.6	37.6	19	2.0	3.4	5.2	19
13	TG	BMI	Uric Acid	Glucose		Non-HDLc		213	21.4	10.8	6.1	17.9	52.9	10	4.8	3.0	8.2	6
14	TG	BMI			HDLc	Non-HDLc		404	10.1	12.1	2.9	17.6	37.1	18	2.0	3.5	5.3	18
15	TG	BMI		Glucose		Non-HDLc		228	**31.5**	**13.7**	6.6	13.7	57.7	5	4.5	2.3	7.1	10
16	TG			Glucose	HDLc			179	25.7	12.3	4.5	21.3	58.3	3	5.7	2.3	8.3	5
17	TG			Glucose	HDLc	Non-HDLc		164	26.9	12.3	4.3	**21.5**	58.4	2	**6.1**	2.6	**9.1**	1
18	TG	BMI		Glucose	HDLc			192	27.2	11.5	4.7	18.8	55.1	8	5.3	3.3	8.8	3

The incidence of the disease is represented as % of disease in cluster 3. Incidence is from KORA FF4 data. The highest incidence of diseases across different models is marked in bold. Rank (metabolic disease) and Rank (cardiovascular disease) are the ranking order of the models based on the highest value of “any metabolic disease” and the highest value of “any cardiovascular disease”, respectively; KORA, Cooperative Health Research in the Region of Augsburg

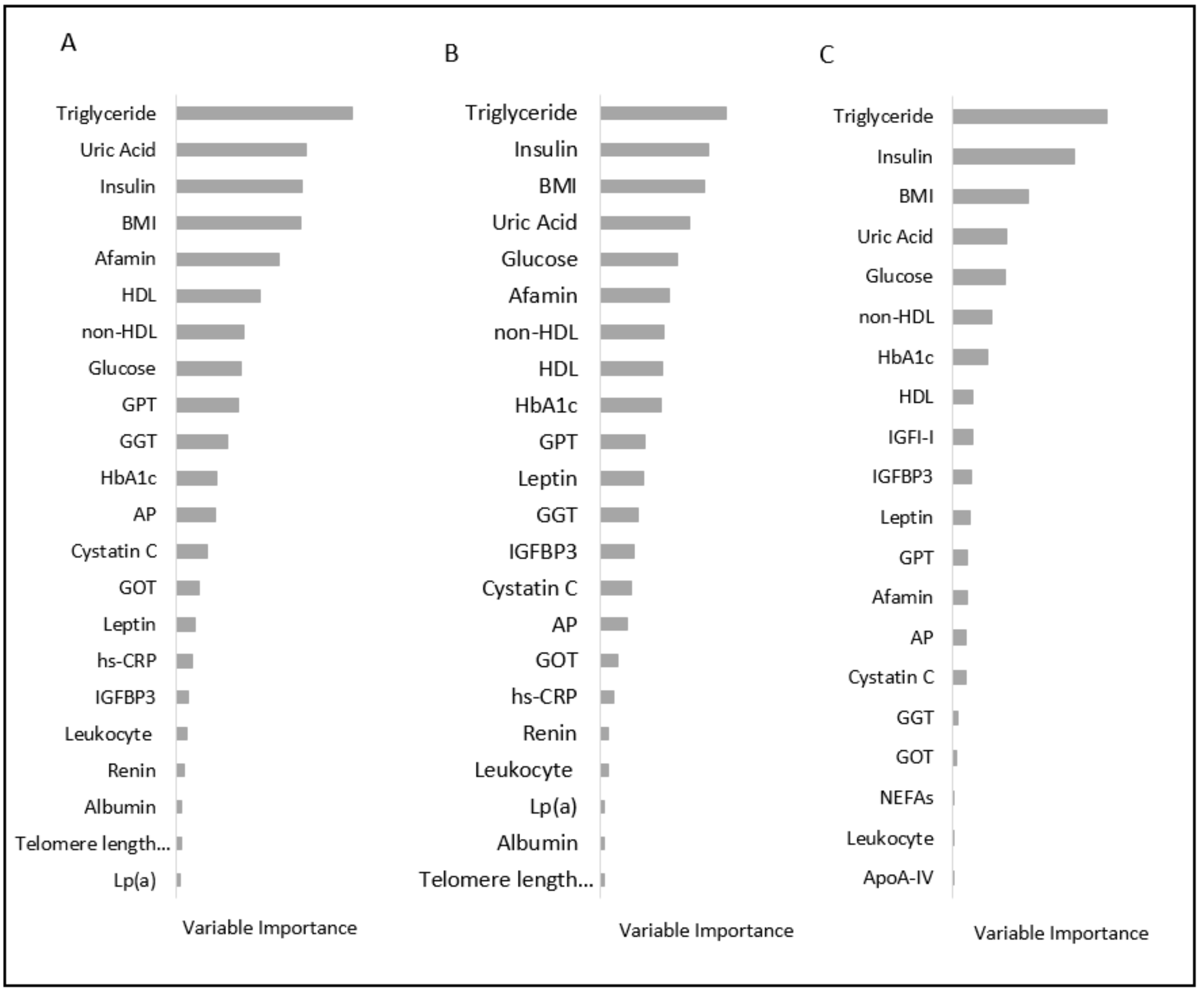

Variable importance from 29 parameters model. Parameters included were: BMI: body mass index; HDLc: high-density lipoprotein cholesterol; non-HDLc: non high-density lipoprotein cholesterol; GGT: gamma-glutamyltransferase; GOT: glutamate-oxaloacetate transaminase; GPT: glutamate-pyruvate transaminase; HbA1c: glycated hemoglobin, hs-CRP: high-sensitive C-reactive protein, AP; alkaline phosphatase; IGF-I: insulin-like growth factor-I ; NEFAs: nonesterified fatty acids; TSH: thyroid-stimulating hormone; IGFBP3: insulin-like-growth-factor-binding-protein-3; FT4: free thyroxine; Lp(a): lipoprotein(a); ApoA-IV: apolipoprotein A-IV; Lp(a): lipoprotein(a); aldosterone; triglyceride; uric acid; insulin; afamin; glucose; cystatin c; leptin; leukocyte; average telomere length in leukocytes (ratio of the telomere repeat copy number to a single copy gene); albumin; renin. Out of 29 variables, only the top 22 variables are shown in the figure.

Figure S1: Variable importance of the 29 clustering parameters using the permutation variable importance (A), cross-validated permutation variable Importance (B), and gradient boosted feature selection method (C).
